# Glial fibrillary acidic protein, neurofilament light, matrix metalloprotease 3 and fatty acid binding protein 4 as non-invasive brain tumor biomarkers

**DOI:** 10.1186/s12014-024-09492-7

**Published:** 2024-06-15

**Authors:** Atefeh Ghorbani, Miyo K. Chatanaka, Lisa M. Avery, Mingyue Wang, Jermaine Brown, Rachel Cohen, Taron Gorham, Salvia Misaghian, Nikhil Padmanabhan, Daniel Romero, Martin Stengelin, Anu Mathew, George Sigal, Jacob Wohlstadter, Craig Horbinski, Katy McCortney, Wei Xu, Gelareh Zadeh, Alireza Mansouri, George M. Yousef, Eleftherios P. Diamandis, Ioannis Prassas

**Affiliations:** 1 Department of Laboratory Medicine and Pathobiology, University of Toronto, Toronto, Canada; 2 Biostatistics Division, Dalla Lana School of Public Health, University of Toronto, Toronto, Canada; 3 Department of Biostatistics, The Princess Margaret Cancer Centre, University of Toronto, Toronto, Canada; 4 Meso Scale Diagnostics, LLC., Rockville, MD USA; 5 Feinberg School of Medicine, Northwestern Medicine, Malnati Brain Tumor Institute of the Robert H. Lurie Comprehensive Cancer Center, Northwestern University, Chicago, IL USA; 6 MacFeeters Hamilton Neuro-Oncology Program, Princess Margaret Cancer Centre, University Health Network and University of Toronto, Toronto, ON Canada; 7 Division of Neurosurgery, Department of Surgery, University of Toronto, Toronto, ON Canada; 8 Department of Neurosurgery, Hershey Medical Center, Hershey, PA USA; 9 Penn State Cancer Institute, Hershey Medical Center, Hershey, PA USA; 10 Laboratory Medicine Program, University Health Network, Toronto, Canada; 11 Department of Pathology and Laboratory Medicine, Mount Sinai Hospital, Toronto, Canada; 12 Lunenfeld-Tanenbaum Research Institute, Mount Sinai Hospital, Toronto, Canada

**Keywords:** Glioma, Biomarkers, Liquid biopsy, Multiplexed electrochemiluminescence (ECL), Differential diagnosis, Glioblastoma, Glial fibrillary acidic protein (GFAP), Neurofilament light (NEFL), Matrix metalloprotease 3 (MMP3), Fatty acid binding protein 4 (FABP4)

## Abstract

**Background:**

Gliomas are aggressive malignant tumors, with poor prognosis. There is an unmet need for the discovery of new, non-invasive biomarkers for differential diagnosis, prognosis, and management of brain tumors. Our objective is to validate four plasma biomarkers – glial fibrillary acidic protein (GFAP), neurofilament light (NEFL), matrix metalloprotease 3 (MMP3) and fatty acid binding protein 4 (FABP4) – and compare them with established brain tumor molecular markers and survival.

**Methods:**

Our cohort consisted of patients with benign and malignant brain tumors (GBM = 77, Astrocytomas = 26, Oligodendrogliomas = 23, Secondary tumors = 35, Meningiomas = 70, Schwannomas = 15, Pituitary adenomas = 15, Normal individuals = 30). For measurements, we used ultrasensitive electrochemiluminescence multiplexed immunoassays.

**Results:**

High plasma GFAP concentration was associated with GBM, low GFAP and high FABP4 were associated with meningiomas, and low GFAP and low FABP4 were associated with astrocytomas and oligodendrogliomas. NEFL was associated with progression of disease. Several prognostic genetic alterations were significantly associated with all plasma biomarker levels. We found no independent associations between plasma GFAP, NEFL, FABP4 and MMP3, and overall survival. The candidate biomarkers could not reliably discriminate GBM from primary or secondary CNS lymphomas.

**Conclusions:**

GFAP, NEFL, FABP4 and MMP3 are useful for differential diagnosis and prognosis, and are associated with molecular changes in gliomas.

**Supplementary Information:**

The online version contains supplementary material available at 10.1186/s12014-024-09492-7.

## Introduction

Glioblastoma (GBM, WHO Grade 4) [1] is the most common primary adult brain cancer. Despite the current standard of care, comprised of maximal safe surgical resection, chemotherapy, and radiation, nearly all GBM inevitably recur, with ultimately fatal outcome. At presentation, clinical and imaging indices alone are insufficient for definitively distinguishing GBM from other malignant intra-axial brain tumors, such as astrocytomas, oligodendrogliomas, brain metastases and primary central nervous system lymphomas (PCNSL) [2]. This is an important unmet clinical need since the optimal management of each disease is different. Differential diagnosis necessitates direct tissue acquisition through a neurosurgical procedure. Unfortunately, surgery poses significant risks. Even minimally invasive stereotactic brain tumor sampling has 4–7% risk of major morbidity and 3% mortality. Such complications can increase hospitalization costs by as much as 10% in a disease that is already known to have a significant financial impact on healthcare, the patients, and their families. Given that non-invasive stereotactic radiosurgery has emerged as a viable option for most brain metastases, and that the management of PCNSL is strictly based on chemotherapy and radiation, the added risk of potentially unnecessary surgery should be avoided. In addition, only a small proportion of resected tumors are used for histological testing, which is complicated with inter-observer variability, with as many as 12% of tumors being misdiagnosed [2]. Due to significant intra-tumor heterogeneity [3], these surgical samples also reflect poorly the entire molecular landscape of the tumor, potentially missing detection of currently established prognostic markers (*e.g., IDH* mutation and *MGMT* promoter hypermethylation) [4–6] and limiting our ability to identify other viable targetable mutations.

In the nearly five decades since randomized, controlled trials first established the benefit of cranial irradiation and carmustine in patients with GBM, the overall survival for afflicted patients has improved by no more than four months [1]. Only two new therapeutic agents have received FDA approval for patients with GBM: one (temozolomide) [4, 5] because it is a less toxic and easier-to-use alkylating agent, and one (bevacizumab) [7] because it improves quality of life but not survival. Although the Tumor Cancer Genome Atlas (TCGA) established three major GBM subtypes, based on transcriptional analysis, this has had limited clinical applicability. Thus far, only *IDH* mutation and *MGMT* promoter methylation have established prognostic value [6]. Establishing additional predictive biomarkers for patient stratification strategies for use in developing targeted therapies and identifying determinants of long-term survival of IDH wild-type GBM remain significant challenges.

Tumor heterogeneity [3] influences initial diagnosis and management and remains relevant even during adjuvant therapy, when serial imaging is used to monitor for true tumor recurrence (TTR) *versus* pseudo-progression (PP; ~15% of cases) or radiation necrosis (RN; ~10% of cases) [8]. Despite advances in imaging modalities, pathological tissue assessment remains the gold standard for distinguishing among these entities, implying that a notable proportion of patients may undergo unnecessary surgery for RN or PP. Short interval follow-up MRI is recommended to distinguish between TTR and PP [9, 10]. Unfortunately, it is not uncommon to observe rapid progression of disease in cases of TTR, at which point enrolment into clinical trials is not feasible due to advanced disease. Thus, it behooves us to develop approaches that help avoid unnecessary surgery and tailor the specific approach based on tumor prognosis when surgery is necessary. Commonly referred to as liquid biopsy, sampling of proximal fluids has offered valuable insight into various systemic cancers as an alternative to tissue biopsy [11, 12].

Considerable efforts have already been undertaken to discover non-invasive, blood-based biomarkers for brain tumor diagnosis, subclassification, prognosis, and treatment response. This need arises, since the standard of care (imaging) is expensive, restricting frequent appointments, and in a proportion of patients, the interpretation of findings is unclear [8]. A plethora of “liquid biopsy” glioma biomarkers have been proposed in the literature [11]. None of them has yet been thoroughly validated or routinely implemented clinically.

Some reported non-invasive glioma biomarkers include extracellular vesicles (EV) [13]. Plasma EV concentration is higher in GBM compared with healthy controls, brain metastases and extra-axial brain tumors. Circulating tumor DNA (ctDNA) is a pan-cancer, non-specific biomarker for many tumors, including gliomas [11, 14, 15]. ctDNA can be isolated from plasma, urine or CSF and subjected to molecular and other analyses, including DNA amount, methylation status and mutational status. This information is valuable for patient diagnosis, prognostication, and management [11, 15]. The method is promising and is used in clinical trials but is expensive (~$1,500 per sample), slow and technically demanding. The value of ctDNA for early diagnosis is currently debated, especially for brain tumors, which release less ctDNA in the circulation than other tumors [16].

Many other individual serum/plasma or CSF protein or nucleic acid biomarkers have been tried for glioma differential diagnosis, prognosis, and therapy response. Prominent among them are glial fibrillary acidic protein (GFAP) [17–19], neurofilament light (NEFL) [20], laminin-5, fibronectin, Type IV collagen, circulating microRNAs; secreted markers of inflammatory response, namely interleukin-6, tumor necrosis factor-α, interferon-γ and kynurenine; and the proliferation markers human telomerase, reverse transcriptase, and microtubule-associated-protein-Tau [19]. Such studies promise to develop and evaluate a non-invasive panel of secreted biomarkers using liquid biopsy [15, 18, 21, 22] for evaluating disease progression, to accomplish a clinical translation.

Despite the current plethora of candidate glioma non-invasive biomarkers, very few, if any, are used routinely, because of their low clinical sensitivity and specificity, high cost and the lack of evidence that they can contribute to improved patient survival or quality of life. In our previous work, we have undertaken the task of using new technology, the proximity extension assay (PEA) [23], to quantitatively profile for the first time ~ 3,000 proteins in plasma of glioma and other brain malignancies to confirm (GFAP, NEFL) and discover additional glioma biomarkers, which, individually or as a small panel, could assist in glioma patient management [24]. We hypothesized that a small panel of non-invasive serum/plasma biomarkers can aid in the optimal management of patients with brain tumors, in combination with current imaging modalities (CT, MRI).

In this paper, we used a different analytical technology (ultrasensitive electroluminescent immunoassay instead of PEA) and an independent, more diverse, and larger cohort of patients, to validate GFAP, NEFL, matrix metalloprotease 3 (MMP3) and fatty acid-binding protein 4 (FABP4), as non-invasive biomarkers of benign and malignant primary and secondary brain tumors. More specifically, we sought to validate our original findings [24], identify additional associations between plasma biomarker levels and molecular characteristics, explore the possibility of differential diagnosis based on the four biomarkers of interest, establish an association between clinical outcomes and biomarker levels (including overall survival), and explore the possibility of differential diagnosis between GBM and primary and secondary CNS lymphomas.

## Materials and methods

### Experimental design and inclusion criteria

Plasma samples from benign and malignant brain tumors were provided by the Northwestern University Brain Tumor Biobank. Plasma samples from apparently healthy individuals were obtained from volunteers working at the University Health Network (UHN), Toronto, Ontario, Canada. These samples included both sexes. The inclusion criteria incorporated plasma samples that were collected from patients that were histologically- and imaging-confirmed for various malignant and benign tumors before surgical intervention, as well as healthy control individuals. Our protocols were approved by the Institutional Review Boards of Northwestern University and UHN. Since our series of patients represent a retrospective cohort, glioma patients were categorized by using the old and the new WHO classification system, as appropriate [25]. We used these classification systems to compare the current data with our previous discovery data, and in particular, we focused on the new classification system [24]. According to the old WHO classification system, the following categories were included: (N = number of samples in brackets): Glioblastoma multiforme (astrocytoma grade 4; *N* = 71), astrocytoma grade 2 (*N* = 13), astrocytoma grade 3 (*N* = 19), oligodendroglioma, grade 2 (*N* = 10), oligodendroglioma, grade 3 (*N* = 13), pituitary adenoma (*N* = 15), Schwannoma, grade 1 (*N* = 15) meningioma, grade 1 (*N* = 45), meningioma, grade 2 (*N* = 25), metastatic adenocarcinoma to CNS (with primary sites in brackets) (colorectal; *N* = 5) (breast; *N* = 10), (lung; *N* = 10), (melanoma; *N* = 10), primary brain lymphoma (*N* = 9), secondary brain lymphoma (*N* = 7) and normal individuals (*N* = 30). The new classification system [25] is mainly based on molecular indices. The following categories were included (N = number of samples in brackets): GBM, gliomas with wild-type IDH (*N* = 77), gliomas with IDH mutant and no 1p19q co-deletion (astrocytomas; *N* = 26), gliomas with mutant IDH and 1p19q co-deletion (oligodendrogliomas; *N* = 23). For each analysis in the Results section, we indicate which categories of patients and classification systems were used. The samples were analyzed in a randomized fashion and the code was broken after the analysis was completed.

Although power calculations were not performed, we decided to increase the power of the statistical analyses, in some cases by combining patients with astrocytoma grades 2 and 3 (*N* = 32) and oligodendrogliomas grades 2 and 3 (*N* = 23). In other analyses, we combined all benign tumors together (pituitary adenomas, Schwannomas grade 1, meningiomas grade 1, and meningiomas grade 2) (*N* = 100).

### Statistical analysis

Patient characteristics were summarized using descriptive statistics: tallies are presented with number (%) for categorical variables. Means (with standard deviations, sd) and medians (with intra-quartile ranges, IQR) are reported for continuous attributes.

Logarithmic transformations: Histograms of the four immunoassay-derived plasma protein levels revealed skewed distributions. Consequently, log transformations have been applied to the biomarker values to obtain near-normal distributions.

Comparison of Olink and immunoassay data: The original Olink assay data for the four candidate biomarkers of interest, presented in our previous manuscript [24], were compared with the Meso Scale Discovery® (MSD) immunoassay data for 50 samples with both data available (Supplementary Fig. 2). Scatterplots were drawn, comparing the protein concentrations between the two methods. Spearman’s rank correlation was calculated as an indicator of the strength of the agreement, with a high correlation coefficient for each marker (Supplementary Fig. 2). The immunoassay from MSD had a lower range for GFAP and NEFL, and thus, saturation was observed, which lowered the r value to a still acceptable value (*r* = 0.91, *r* = 0.90 for GFAP and NEFL respectively).

Validation of data of previous findings: We replicated our original findings [24] (Olink data) with the present data (immunoassay) to identify gliomas and meningiomas using FABP4 and GFAP levels utilizing (1) thresholds that achieve 100% specificity, (2) using logistic regression. Because the immunoassay and Olink values do not use the same analytical units, we identified method-specific thresholds. We then evaluated the performance (sensitivity, specificity, area under the curve (AUC)) of the models based on these thresholds.

Assay reproducibility: Since we had only four patients with repeat analyses (due to plasma volume depletion), no statistical analysis was performed for assay reproducibility, but the repeat observations were plotted onto the protein concentration distributions, to provide a visual semi-quantitative assessment of the assay precision.

### Biomarkers and patient characteristics

Exploratory analyses were conducted to determine if biomarker levels were associated with age, sex, or ethnicity for the entire sample (*n* = 291) as well as the subset of patients with glioma (*n* = 126). Scatterplots and Spearman correlation coefficients were calculated for each biomarker according to age. For sex and ethnicity, ${\chi }^{2}$ tests were performed.

### Genetic variables and biomarker concentrations

Genetic mutation status was available from the Northwestern University Biobank for the subset of patients with glioma (*n* = 126). There was a single patient with EGFR VIII status who was not included in the analysis. In our study, *t* tests were used to determine if there were differences in biomarker concentrations between the following genetic variants: IDH1(wild-type vs. mutant), ATRX expression (retained or lost), P53 NGS (wild-type or mutant), EGFR (wild-type vs. mutant), MGMT promoter methylation (positive or negative), TERT promoter (wild-type or mutant), CDKN2A (wild-type or lost), 1p19q co-deletion (negative or positive) and NF1 (wild-type or mutant). Boxplots were created to display the differences in numerical or categorical variables.

### Diagnosis and grade and biomarker concentrations

Plots of the biomarker concentrations as a function of diagnostic category and WHO grade were created. For a subset of glioma patients, multivariable linear regression models were used to explore the effect of diagnostic category and WHO grade on biomarker concentrations.

### Association between biomarker concentration and survival

Since the four biomarker concentrations vary by diagnostic category, and diagnosis is predictive of overall survival, an exploration of the association between biomarker concentration and survival was conducted within diagnostic groups for three diagnostic categories: GBM (wild-type IDH), meningioma and secondary tumors (tumors that metastasized to the brain); these categories were chosen because each subgroup had at least five recorded deaths. Initially, Cox proportional hazards models were fit, but the proportional hazards assumption was not met for several biomarker/diagnostic combinations; consequently, simple Kaplan-Meier survival curves were drawn, using the median as cut-off.

All analyses were conducted using the R statistical programming language version 4.2.2 [26].

### Meso Scale Discovery (MSD) assays

MSD’s combination of electroluminescence and multianalyte immunoassay technology provides exceptional sensitivity and multiplex functionality, making it a highly useful analytical immunoassay system. For more descriptions of the MSD assays and their applications, please refer to our previous publications [23, 27–29] and the manufacturer’s website (www.mesoscale.com). The MSD assay characteristics that apply to the four assays used are as follows: The analytical sensitivity (limit of detection, LOD) is 2–4 pg/mL. Precision is generally < 15% and all plasma samples were measured in duplicate. Concentrations above the upper end of the assay range (upper limit of quantification, ULOQ) were assigned the ULOQ concentration. The assays are devoid of any known interferences.

### Demographic variables

Supplementary Table 2 summarizes the patient demographic characteristics. The subject information was blinded prior to analysis. The age, sex, and ethnicity of the 291 patients was recorded.

## Results

### Biomarker distributions

The distributions of plasma concentrations of the four candidate biomarkers GFAP, NEFL, MMP3 and FABP4 among all patients are shown in Supplementary. All proteins have skewed distributions (not shown) but after logarithmic transformation, the distributions become near normal. 

**Fig. 1:**
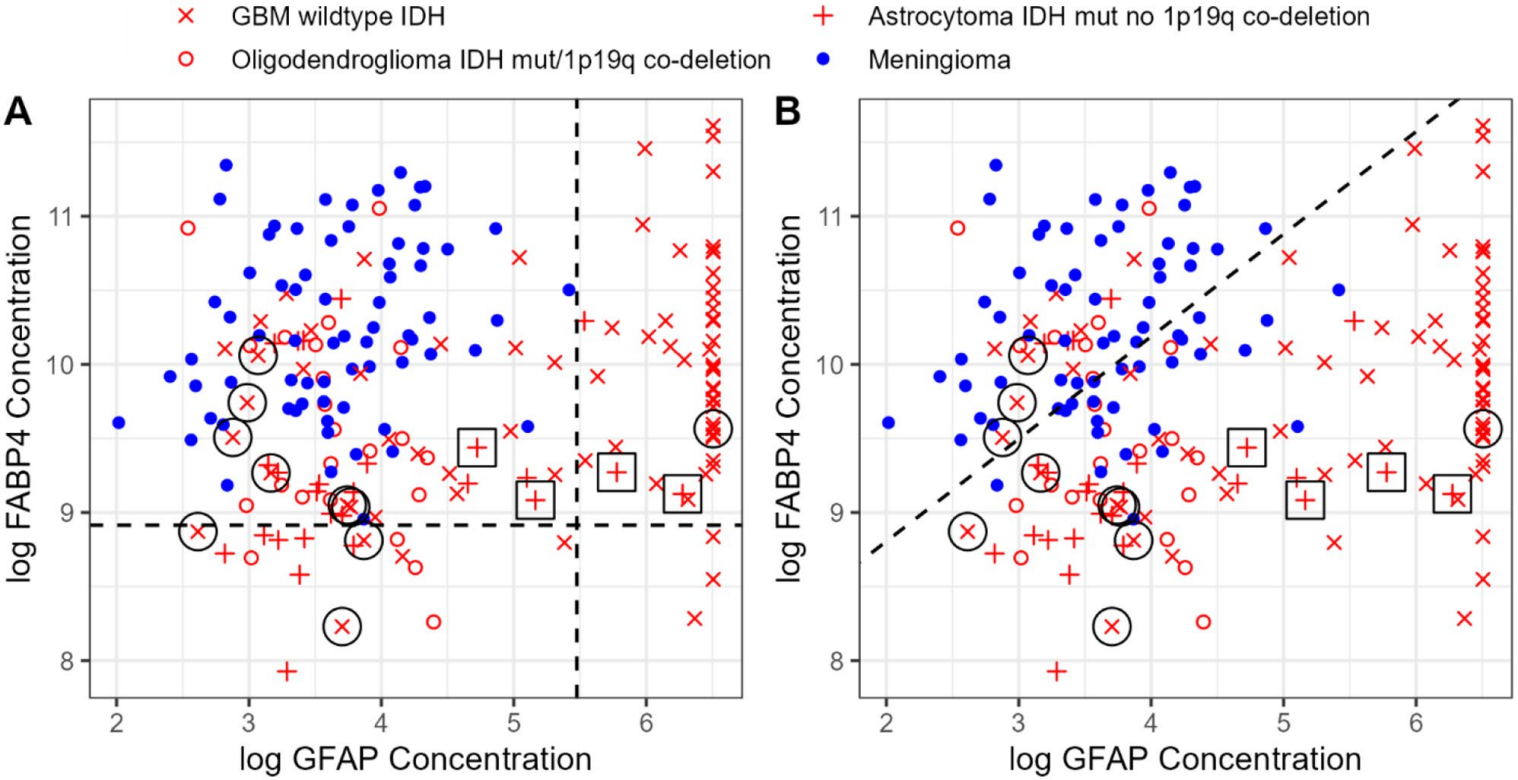
Separation of various brain tumors by using a combination of plasma GFAP and FABP4. Our preliminary findings [22] have been replicated using MSD immunoassay, instead of Olink PEA technology. **(A)**: 100% specificity threshold. All patients with GFAP < 226 pg/mL (vertical dotted line) and FABP4 > 7,736 pg/mL (horizontal dotted line) had meningioma (blue dots), with 53% sensitivity to detect glioma. **(B)**: Logistic regression model with 64% specificity and 84% sensitivity for meningiomas and gliomas, respectively (shown is the separating dotted line). Cases for whom the diagnosis has changed under the new WHO classification are highlighted with circles, indicating a GBM with wild-type IDH, previously categorized as astrocytoma. Squares indicate astrocytomas with IDH mutation, previously [22] classified as GBM. For more discussion see text

### Comparison of Olink PEA assays and MSD immunoassays

In our previous biomarker discovery investigation, we used Olink analytical technology (PEA) to quantify plasma GFAP, NEFL, MMP3 and FABP4, in addition to another 3,000 proteins. The concentrations of all proteins are expressed in relative quantification (NPX) values [23]. In the present validation study, we quantified the 4 proteins in plasma with specific, sensitive, precise, and quantitative MSD® multiplexed immunoassays. For 50 plasma samples, we paired Olink and immunoassay results for comparison (Supplementary Fig. 2).

The Spearman rank-order correlation coefficients are shown in the bottom-right corner of each plot. The agreement is generally good, except when GFAP and NEFL values exceed the ULOQ and were assigned the ULOQ concentration.

### Validation of our previous findings

We previously found [24] that high plasma GFAP was associated with GBM, low GFAP and high FABP4 were associated with meningiomas and low GFAP and low FABP4 were associated with astrocytomas. For this independent validation study, the samples considered were from patients with similar final diagnoses in the new set of patients (*N* = 196) as shown in Supplementary Table 1 (we used pre 2021 and post 2021 WHO glioma classification).

Analysis of the data was accomplished with two different methods as follows:

#### Method 1

Analytical parameter cutoffs were set at 100% specificity for separating meningiomas from gliomas. As shown in Fig. 1 panel A, all patients with meningiomas had GFAP < 226 pg/mL and FABP4 > 7,736 pg/mL. The two-marker (GFAP/FABP4) combination model performance was as follows: at 100% specificity (where all 70 meningiomas were correctly predicted to be meningiomas), the sensitivity for glioma detection was 53%, the positive predictive value (PPV) for meningioma detection was 1.00, the negative predictive value (NPV) was 0.54, and the accuracy was 70%. Oligodendrogliomas grade 2 and 3 and astrocytomas grade 2 and 3, in general, exhibited low FABP4 and low GFAP values. These data are in accordance with those of our previous, smaller study [24].

#### Method 2

Logistic Regression. A logistic regression model was fitted combining GFAP and FABP4 (Fig. 1 panel B). With this model, at 64% specificity (among 70 meningiomas 25 were misclassified as gliomas), the sensitivity for glioma detection is 84% (20 gliomas were misclassified as meningiomas). The PPV was 0.81, the NPV was 0.69, and the overall accuracy was 77%.

These data are in accordance with our previous findings [24], summarized as follows: High GFAP was associated with GBM, low GFAP and high FABP4 were associated with meningiomas grade 1 and 2 and low GFAP and low FABP4 were associated with astrocytomas grade 2 and 3 (mutant IDH, no 1p19q co-deletion) and oligodendrogliomas grade 2 and 3 (mutant IDH with 1p19q co-deletion).

### Assay reproducibility

The manufacturer-stated analytical reproducibility of the four utilized MSD assays is < 15%. We have limited data (only two replicates for plasma samples from 4 patients) to systematically examine reproducibility, due to patient sample depletion. Consequently, no extensive reproducibility statistics have been calculated. Instead, for the four biomarkers of interest, the available replicates are shown by super-imposing them on the full cross-sectional data (all values are log-transformed). The color of the dots and the y-axis positioning are the same for each patient with multiple assay values (see Supplementary Fig. 3). In most cases with available duplicates, the assay values are similar, and the y-axis positioning are the same for each of the four patients with duplicate assay values. Where only a single dot is visible, the values of both replicates are identical. For most samples with duplicate values, the concentrations are similar. Due to the small number of replicates (the reason being plasma sample depletion) statistical analyses were not done. For more comments see text.

### Age and protein concentrations

All 291 patients with age information were included. The data are presented in Supplementary Fig. 4. Age is positively, but weakly, correlated with all four protein concentrations. The Spearman correlation coefficient ranged from 0.26 to 0.47 and it is shown on top of each graph.

### Effect of patient sex

The effect of patient sex on plasma protein concentrations are presented in Supplementary Tables 3 and graphically in Supplementary Fig. 5. Three proteins (GFAP, MMP3, NEFL) are higher in males and one protein (FABP4) is higher in females. All patients with sex information were included.

### Effect of ethnicity

The effect of ethnicity on plasma biomarker concentrations is shown in Supplementary Tables 4 and graphically in Supplementary Fig. 6. In general, Blacks have the highest plasma biomarker values and White Hispanics the lowest, but the differences are small.

### Genetic variables and biomarker concentrations

**Fig. 2:**
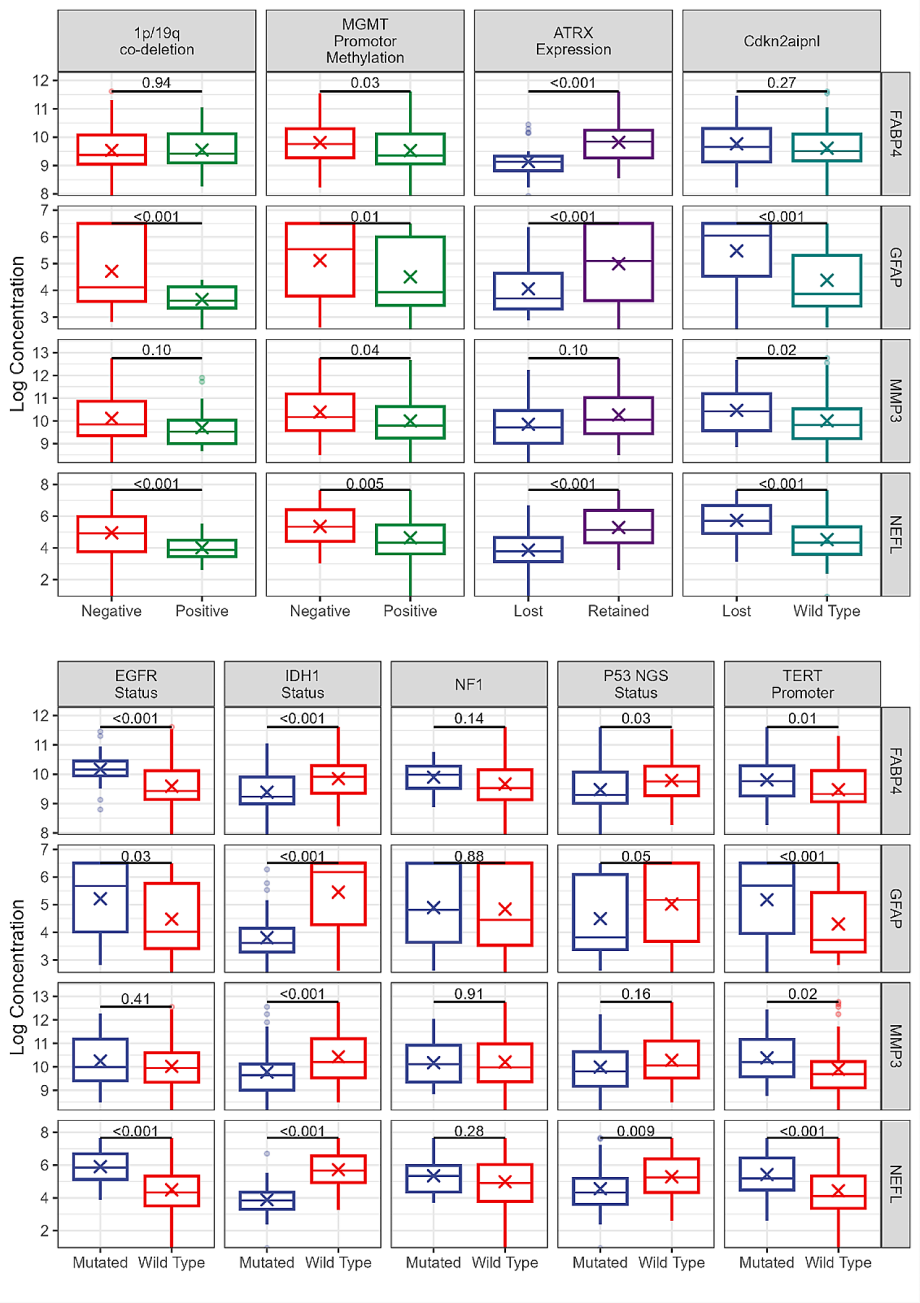
Comparison of genetic and proteomic markers. Genetic markers of gliomas and their relationship to plasma concentrations of the four biomarkers shown (FABP4, GFAP, MMP3, NEFL). The figure shows the median (horizontal lines), and interquartile range. Means are indicated with an ‘x’. Significance levels for independent *t* tests are shown at the top of each plot and have not been adjusted for multiple comparisons. Despite several differences indicating statistical significance, these differences are generally small. For more details of patient genetic status see also Supplementary Table 5. For GFAP, the most important differences (P = < 0.01) were between GFAP and IDH1 status, ATRX expression, MGMT promoter methylation, TERT promoter mutation, and CDKN2A/B p16 and 1p/19q co-deletion

We used a retrospective cohort of samples from a biobank, collected over many years, and not all genetic changes are reported for all patients. Values not reported were re-coded as missing. For EGFR status there was a single patient with EGFR VIII; this patient was not included in the comparisons. Despite several tests indicating statistical significance between protein concentrations and genetic changes, the magnitude of the differences is relatively small. (See Supplementary Tables 5 and graphically in Fig. 2, below). Note that these data have not been adjusted for multiple comparisons, because our main purpose was to determine if these factors should be incorporated into a diagnostic model.

### Descriptive plots

**Fig. 3:**
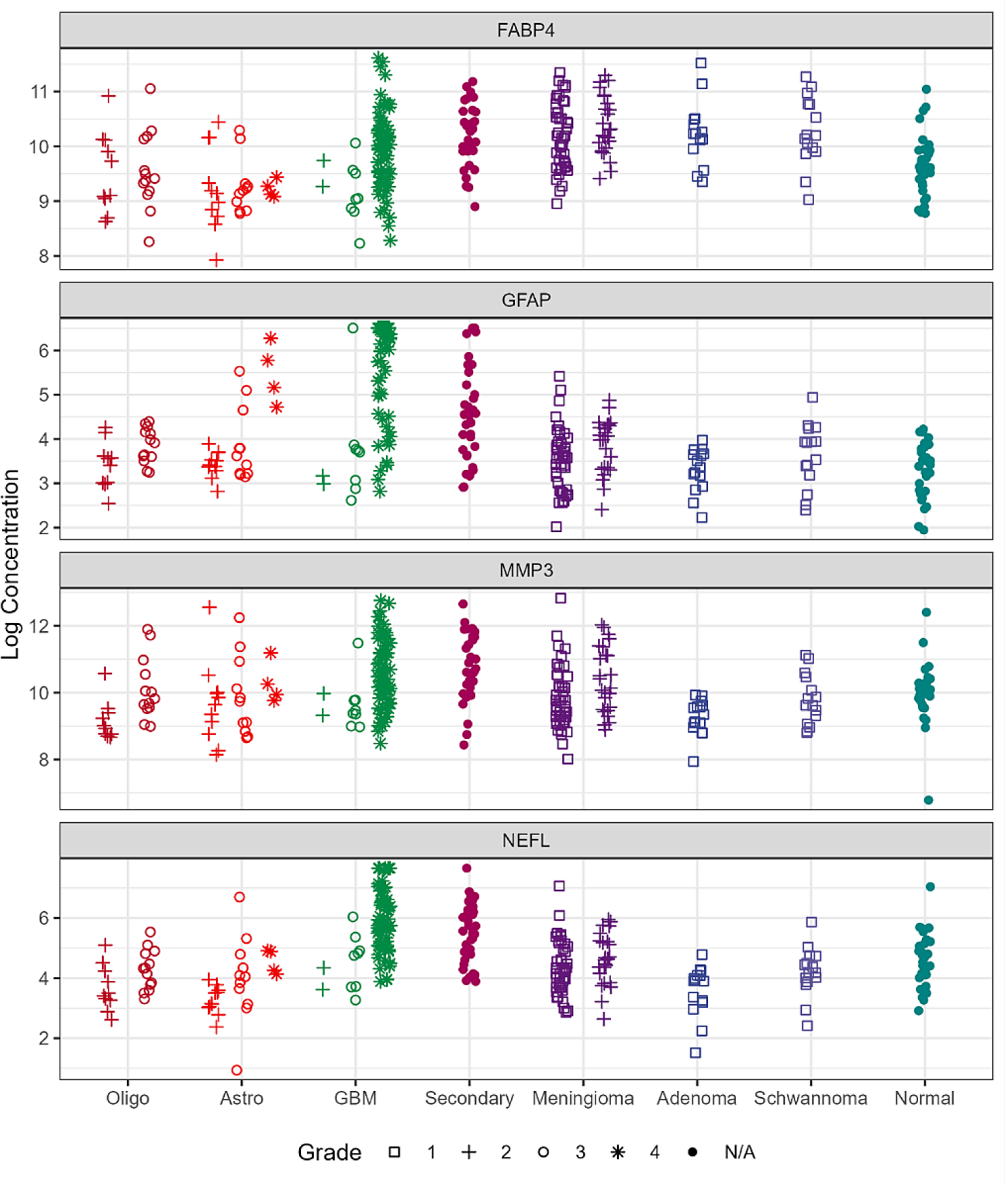
Scattergrams of the four protein plasma concentrations (FABP4, GFAP, MMP3, NEFL) by diagnostic groups. Oligo = Oligodendroglioma IDH mut/1p19q co-deletion, Astro = Astrocytoma IDH mut, no 1p19q co-deletion, GBM = GBM wild-type IDH, Secondary = secondary tumors, Adenoma = pituitary adenoma, Schwannoma = Schwannoma, grade 1. For each diagnostic group the patients are stratified by WHO grade along the x-axis with different symbols. For more comments see text. Number of samples per category can be found in Supplementary Tables 1 and 2

In Fig. 3, we present scattergrams showing the four biomarker concentrations for each patient group, and according to tumor grade. There is significant overlap between biomarker values for all shown patient groups. In general, the highest levels of the biomarkers are seen in GBM, except FABP4 which is higher in meningiomas vs. gliomas.

### WHO grade and glioma subclass

In our sample set, WHO grade is associated with the diagnostic category, with most grade 4 patients diagnosed with GBM wild-type IDH (Supplemental Table 6). We do not have sufficient sample sizes to perform sub-group analyses within grade or diagnostic category, to properly investigate whether biomarker levels are associated primarily with disease type or with disease severity. Instead, we have attempted to estimate the relative importance of diagnosis and grade on biomarker levels by undertaking multiple linear regression to estimate protein levels from diagnostic category and WHO grade. For each of the four biomarkers of interest, we fit a multivariable model with both diagnosis and grade, and separate models for each predictor. The unique contribution to R^2^ was then equal to total R^2^ less R^2^, without the predictor of interest. Supplemental Table 7 summarizes the findings and indicates that WHO grade is the most important predictor of protein levels in this sample. This has important implications for future work; longitudinal studies of early-stage patients will be necessary to determine if protein levels are diagnostic, prognostic or both.

The effect of WHO grade on the values of the four biomarkers is further shown graphically in Fig. 3.

**Fig. 4:**
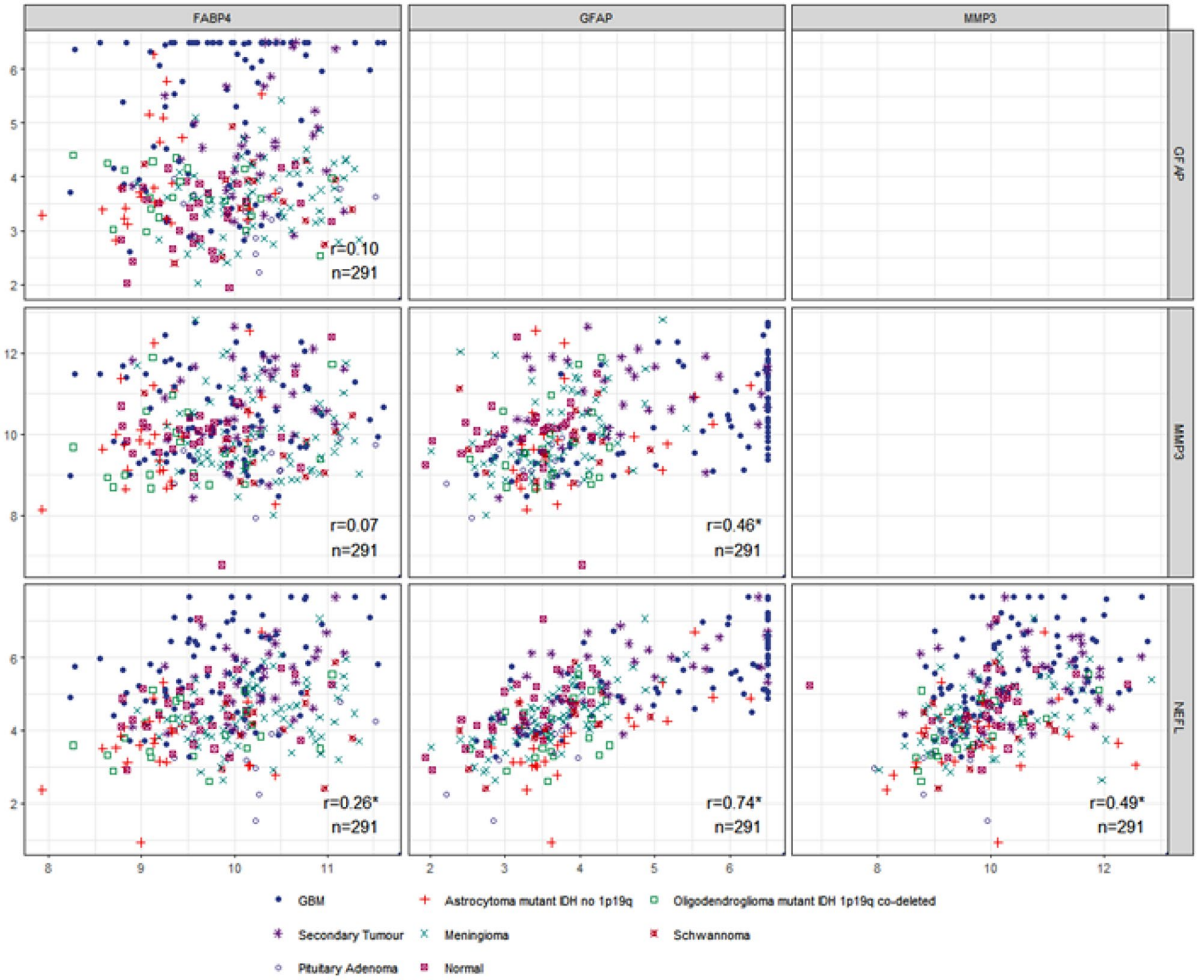
Correlation between the four markers for various patient groups. Correlation between the four biomarkers for GBM (*n* = 77), astrocytoma with mutant IDH no 1p19q co-deletion (*n* = 26), oligodendroglioma mutant IDH with 1p19q co-deletion (*n* = 23), secondary tumors (*n* = 35), meningioma (*n* = 70), Schwannoma (*n* = 15), pituitary adenoma (*n* = 15) and normal controls (*n* = 30). Spearman correlation is shown on the right bottom corner of each plot. NEFL shows the strongest correlation with GFAP (*r* = 0.74). The reported Spearman correlations were calculated by including all patient groups

We investigated the correlations of each biomarker with the other three biomarkers in the various diagnostic groups. An example is shown in Fig. 4, which includes all diagnostic groups. For all comparisons (not all data are shown) the strongest correlations were between NEFL and GFAP, with Spearman correlation coefficients (r) in the range of 0.7. For the other comparisons between the biomarkers the Spearman correlation coefficient was lower (Fig. 4).

### Survival and biomarker concentrations

**Fig. 5:**
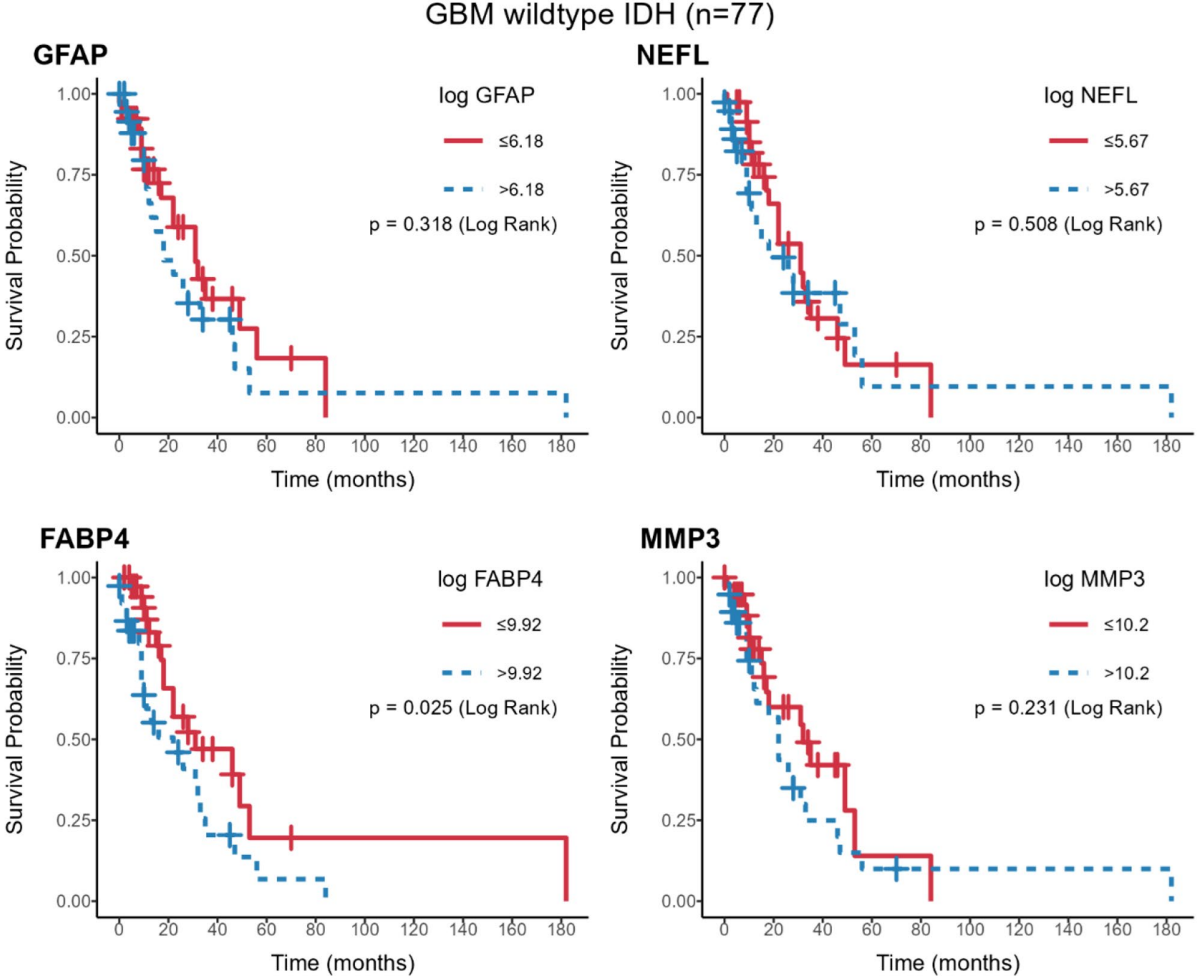
Survival analysis. Survival analysis (Kaplan-Meier plots) of patients with GBM, divided into high (blue crosses) or low (red crosses) plasma GFAP, NEFL, MMP3 and FABP4 level. The median of each protein concentration was used as a cut-off. Cut-offs are shown after log transformation, along with the p value, calculated by the log-rank test. Only patients with high FABP4 have significantly lower survival (*p* = 0.025)

To explore associations between plasma protein levels and overall survival we examined survival status within each diagnostic category. While this limited the sample size, it also removed confounding due to the diagnostic category (the latter is independently associated with survival). Initially, Cox proportional hazards models were fit to investigate the effect of protein levels on survival, but the proportional hazards assumptions were often violated. So, for this exploratory work, which should be considered hypothesis-generating, we chose to examine Kaplan-Meier curves, splitting the sample at the median protein level. Analysis was limited to the three patient groups with a minimum of five observed deaths: GBM wild-type IDH, meningioma and secondary tumors. Data for GBM are shown in Fig. 5 and for the other two groups in Supplementary Figs. 7 and 8.

### Longitudinal plots

For 24 patients we had plasma collected at both diagnosis and recurrence. We compared these two values to obtain a preliminary estimate of their changes during progression. We found that on average, values tend to be higher at recurrence, but not for all patients. For FABP4 and MMP3 no changes > 50% in either direction were seen. For GFAP one patient had a > 50% decrease at recurrence and 3 patients had > 50% increase at recurrence. For NEFL, four patients had > 50% increase at recurrence (Supplementary Fig. 9). All other patients had changes of < 50% in magnitude.

## Discussion

One of the unmet clinical needs in the area of brain tumors is the discovery and validation of non-invasive biomarkers and other technologies which can assist in optimal patient management. These tools could help in disease differential diagnosis, prognosis, monitoring the success of treatments, and facilitate early inclusion of patients in clinical trials, as new therapies become available. In addition to the valuable, but expensive and slow, imaging technologies, methods based on liquid biopsy, utilizing serum or plasma, could have important complementary clinical utility. Recently, we used a new, powerful proteomic technology, PEA (Olink Proteomics), to simultaneously quantify about 3,000 plasma proteins in patients with gliomas and meningiomas (as controls) [24]. Among all proteins, a few had discriminatory potential between gliomas and meningiomas. In this paper, we validated our previous findings for the 4 most promising candidate biomarker proteins (GFAP, NEFL, MMP3 and FABP4) and extended our previous observations by using a larger and more diverse set of well-characterized samples from benign and malignant primary and secondary brain tumors.

In our previous work we analyzed plasma samples by the PEA technology [23] collected from glioma patients of various WHO grades before therapy initiation and compared the results with age- and sex-matched patients with meningiomas. In the current work, we used an orthogonal quantification technology (MSD electroluminescence immunoassays), to simultaneously quantify the four proteins in plasma, (GFAP, NEFL, MMP3 and FABP4), and examined their value as diagnostic and prognostic markers. The MSD assays used are more economical than PEA, sensitive, specific, quantitative, and more precise, requiring small plasma volumes, and they are ideal for a large-scale validation of biomarkers. The four selected biomarkers for validation were chosen for their performance in the discovery phase and their link to brain tumors.

The four candidates, GFAP [30–34], NEFL [35, 36], MMP3 [37–39] and FABP4 [40–43] have previously been associated with brain tumors in small studies and have also been shown to be elevated in plasma and cerebrospinal fluid (CSF) of patients with other brain disorders, including traumatic brain injury, neurodegeneration, multiple sclerosis, etc [44–48]. The non-specificity of our markers for brain tumors is a clear disadvantage, but the clinical presentation of these brain disorders is sufficiently different from brain tumors. Additionally, our biomarkers will likely find more clinical applicability during management of already diagnosed brain tumors.

In this study, we found several new associations between patient clinicopathological features and the four candidate plasma biomarkers. As in the previous study, we found that GFAP, as compared to the other three proteins, had the highest discriminatory potential between gliomas and meningiomas. The combination of two markers (GFAP and FABP4) further enhances the discrimination between gliomas and meningiomas (Fig. 1).

The putative functional role of GFAP in astrocytes (the main type of glial cells in the central nervous system (CNS)) was previously reported [46]. GFAP is involved in numerous astrocyte functions. In the early stages of recovery following brain surgery, GFAP increases in response to astrocytic reaction to brain injury [49].

Convincing evidence supports the involvement of GFAP in GBM. Serum GFAP was significantly increased in WHO grade 4 glioma (GBM) and was detected in 63% of all grade 4 patients compared to 13% of healthy controls [31, 50], , suggesting that glioma patients have elevated plasma GFAP, in accordance with our findings. (Fig. 3). Serum GFAP correlates with invasiveness in astrocytomas and high-grade gliomas, compared to lower grade gliomas. Thus, GFAP represents a potential prognostic biomarker and a candidate therapeutic target for gliomas [51].

Another well-known glioma biomarker is NEFL (neurofilament light polypeptide) also known as neurofilament light chain, a potential tumor suppressor [20]. NEFL is involved in a variety of common human cancers such as breast, prostate, and head and neck cancers. Plasma NEFL concentration was higher in patients with CNS tumors with disease in progression versus CNS tumors with stable disease. In addition, plasma NEFL was higher in patients with metastatic solid tumors with known brain metastases than in those with metastatic tumors with no brain metastases [52]. As such, NEFL is also a prognostic marker of brain tumors.

FABP4 is one of ten intracellular small molecular weight proteins that belong to the FABP family and is found in adipose tissue, peripheral macrophages, and microglia but not in normal brain blood vessels, although it has been found in certain endothelial cells or tumor cells in benign and malignant meningiomas. FABP4 could have a role in carcinogenesis in meningiomas by stimulating cell proliferation in a cell type-independent way [41, 42]. In this connection, rapamycin, a well-known inhibitor of the mTOR pathway, which is a master regulator of cell growth and metabolism, inhibits FABP4 production by endothelial cells. FABP4 is expressed in a significantly higher percentage of GBMs in comparison to both normal brain tissues and lower-grade glial tumors. Other data suggest that FABP4 may play a role in angiogenesis associated with GBMs. Another study analyzed FABP4 expression in a cohort of paraffin-embedded meningioma specimens by immunohistochemistry and double immunofluorescence analyses. These results demonstrate that FABP4 is commonly expressed in meningioma vascular endothelial cells while tumor cell expression of FABP4 is primarily observed in anaplastic meningiomas. A combination of FABP4 immunostaining with histopathologic grading might provide a more accurate prediction of the biological behavior of meningiomas than histopathologic grading alone.

MMP3 plays a role in cell migration. Platelet-derived growth factor receptor-alpha (PDGFR-alpha) induces MMP3 gene expression and increased cell proliferation and cell migration upon stimulation by PDGF. The induction of expression of MMP3 in glioblastoma cells triggers a cascade of gene expression events, resulting in decreased cell adhesion and migration [37].

Among our new findings, we report for the first time the dependence of the plasma concentration of these candidate biomarkers on genetic changes frequently seen in gliomas (Fig. 2). In general, mutations that are associated with better patient prognosis are associated with lower levels of the four biomarkers in plasma. This observation may be clinically useful. Tumors with late-stage disease (grade 4) are usually associated with higher levels of these proteins in plasma (Fig. 3). However, protein levels were not significantly associated with overall patient survival (Fig. 5 and Supplementary Figs. 7 and 8), presumably due to the small number of patients and to the rather short follow-up time. Pairwise plots have shown that among the four proteins, the strongest correlation was seen between GFAP and NEFL. (Fig. 5).

Primary and secondary CNS lymphomas are relatively rare brain tumors, and our lymphoma cohort is rather small and does not allow for definitive conclusions (data not shown). However, we found qualitative evidence that lymphomas demonstrate variable levels of the four proteins, with high overlap between the patient groups (gliomas and lymphomas). From these data (which were not included in this paper) we conclude that these four biomarkers cannot discriminate primary or secondary lymphomas from GBM, even if the levels of GFAP are, in general, higher in GBM in some cases.

Patients from whom we had two samples, at diagnosis and at relapse (Supplementary Fig. 9), showed a trend for increase of the four biomarkers with time. Although we could not confidently conclude from these limited data, we speculate that changes in these biomarkers with time, and their possible correlation with tumor progression, may qualify these proteins as non-invasive, cheap, and fast tools for monitoring patients with brain tumors, including assessment of the effectiveness of new therapeutic agents. To this end, we are currently prospectively recruiting patients to establish the monitoring value of these four biomarkers in brain tumors.

### Limitations

Our study is retrospective and the provision of the samples by a Biobank may be associated with sample collection and storage bias. Our primary and secondary brain lymphoma cases are few due to the relative rarity of these conditions. Our biomarkers may perform well for the intended application (aid in diagnosis and management) but they may not contribute towards better patient survival, quality of life or selection of treatments.

### Electronic supplementary material

Below is the link to the electronic supplementary material.

Supplementary Material 1

## Data Availability

The excel file with the concentrations of the four biomarkers, as well as the anonymized clinical information are available by request from the corresponding author (EPD).

## Abbreviations

GBM: Glioblastoma
PCNSL: Primary Central Nervous System Lymphomas
IDH: Isocitrate Dehydrogenase
MGMT: O(6)-Methylguanine-DNA Methyltransferase
TCGA: Tumor Cancer Genome Atlas
TTR: True Tumor Reoccurrence
PP: Pseudo-Progression
RN: Radiation Necrosis
EV: Extracellular Vesicles
ctDNA: circulating tumor DNA
GFAP: Glila Fibrillary Acidic Protein
NEFL: Neurofilament Light
PEA: Proximity Extension Assay
MMP3: Matrix Metalloprotease 3
FABP4: Fatty Acid-Binding Protein 4
UHN: University Health Network
IQR: Intra-Quartile Range
MSD: Meso Scale Discovery®
AUC: Area Under The Curve
EGFR: Epidermal Growth Factor Receptor
ATRX: Alpha-Thalassemia mental Retardation X-linked
NGS: Next Generation Sequencing
TERT: Telomerase Reverse Transcriptase
CDKN2A: Cyclin-Dependent Kinase Inhibitor 2 A
NF1: Neurofibromatosis type 1
LOD: Limit Of Detection
ULOQ: Upper Limit Of Quantification
NPX: Normalized Protein Expression
PPV: Positive Predictive Value
NPV: Negative Predictive Value
CSF: Cerebrospinal Fluid
mTOR, PDGFR-alpha: Platelet-Derived Growth Factor Receptor- alpha
