# Supplementary material for: Glial fibrillary acidic protein, neurofilament light, matrix metalloprotease 3 and fatty acid binding protein 4 as non-invasive brain tumor biomarkers
Source: Clin Proteomics. 2024 Jun 15;21:41. doi: 10.1186/s12014-024-09492-7 (PMC11179213; doi:10.1186/s12014-024-09492-7)
Supplement: Supplementary file 1 — Supplementary Material 1 [file 12014_2024_9492_MOESM1_ESM.docx]

Supplementary Materials for

**Glial fibrillary acidic protein, neurofilament light, matrix metalloprotease 3 and fatty acid binding protein 4 as non-invasive brain tumor biomarkers**

Atefeh Ghorbani *et al*.

*Corresponding author. Email: [diamandis@lunenfeld.ca](mailto:diamandis@lunenfeld.ca) or yprassas@gmail.com

**This PDF file includes:**

Supplementary Figures 1 to 9

Supplementary Tables 1 to 7


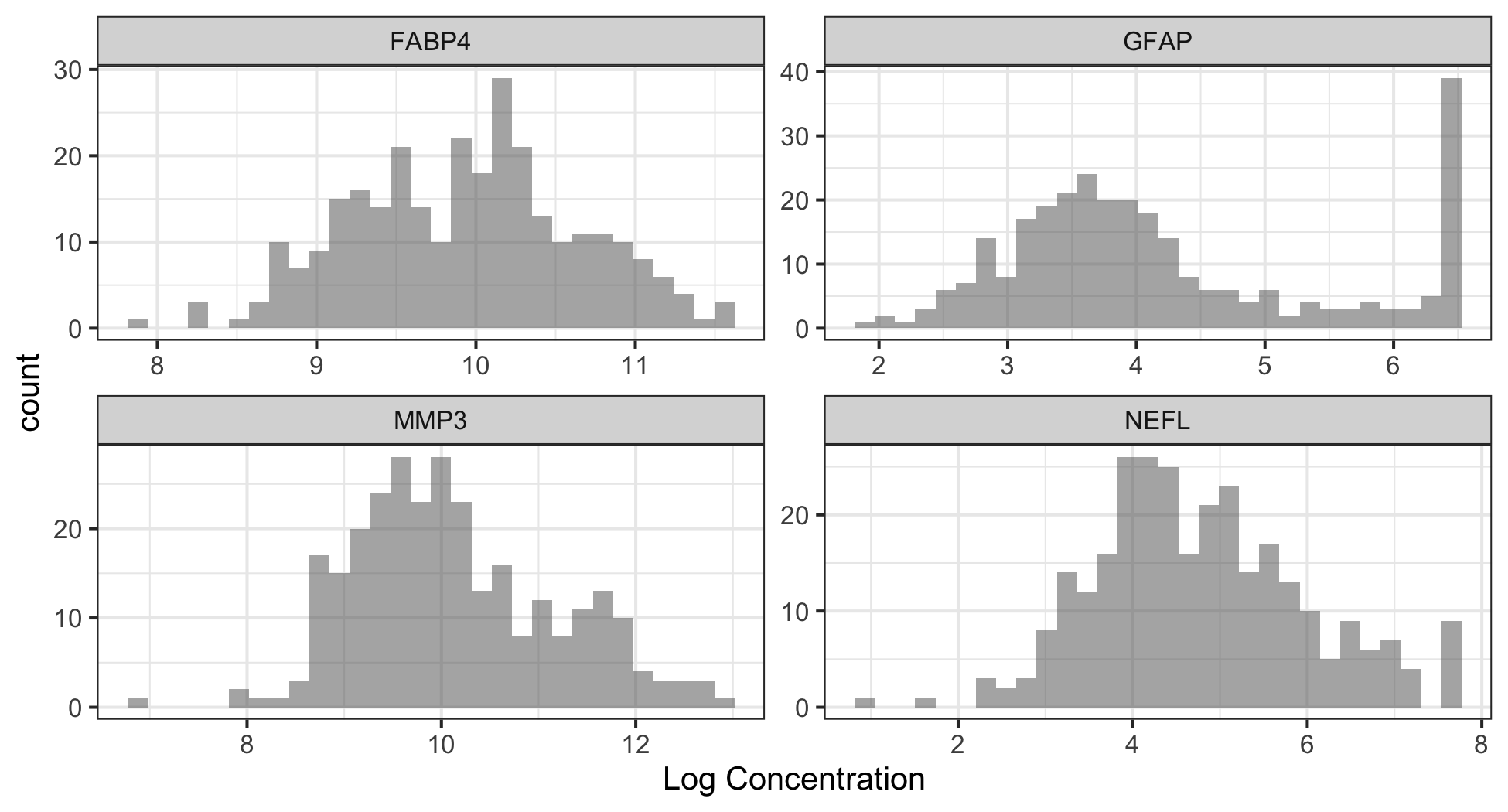


**Supplementary Figure 1**. Distribution of the four proteins (FABP4, GFAP, MMP3, NEFL), in all measured plasma samples after logarithmic transformation (MSD assays). Raw protein concentrations were in pg/mL.


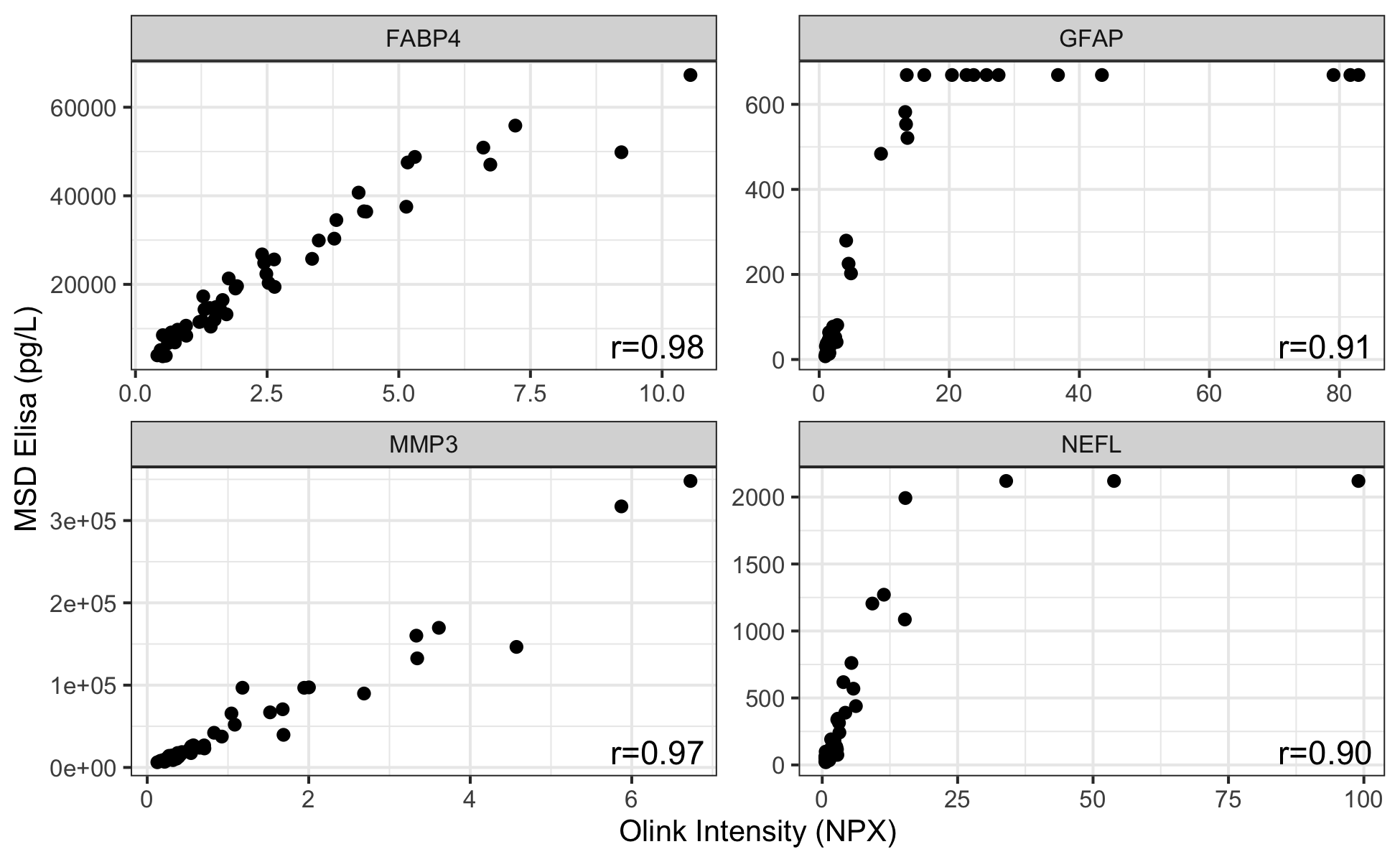


**Supplementary Figure 2**. Comparison of Olink (x-axis) and MSD assays (y-axis) for the four proteins shown, for 50 plasma samples. The Spearman correlation coefficient, r, is shown on the bottom-right corner. For additional commends and definitions see text

*
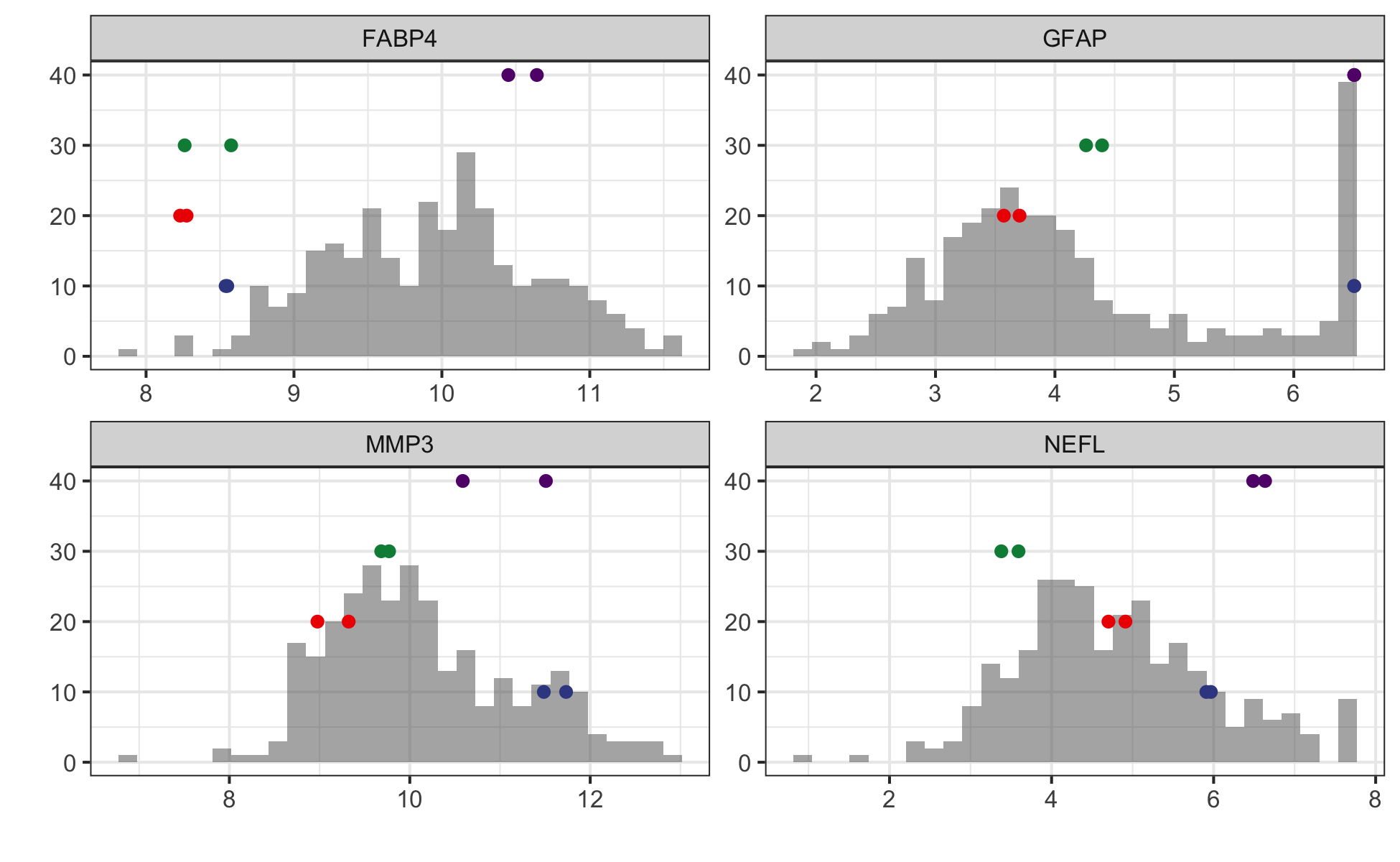
*

**Supplementary Figure 3**. For the four biomarkers of interest (FABP4, GFAP, MMP3, NEFL), duplicate measurements are shown by superimposing them on the full cross-sectional data (all values are log-transformed). The color of the dots and the y-axis positioning are the same for each of the four patients with duplicate assay values. Where only a single dot is visible, the values of both replicates are identical. For most samples with duplicate values, the concentrations are similar. Due to the small number of replicates (the reason being plasma sample depletion) statistical analysis was not performed. For more comments see text.

*
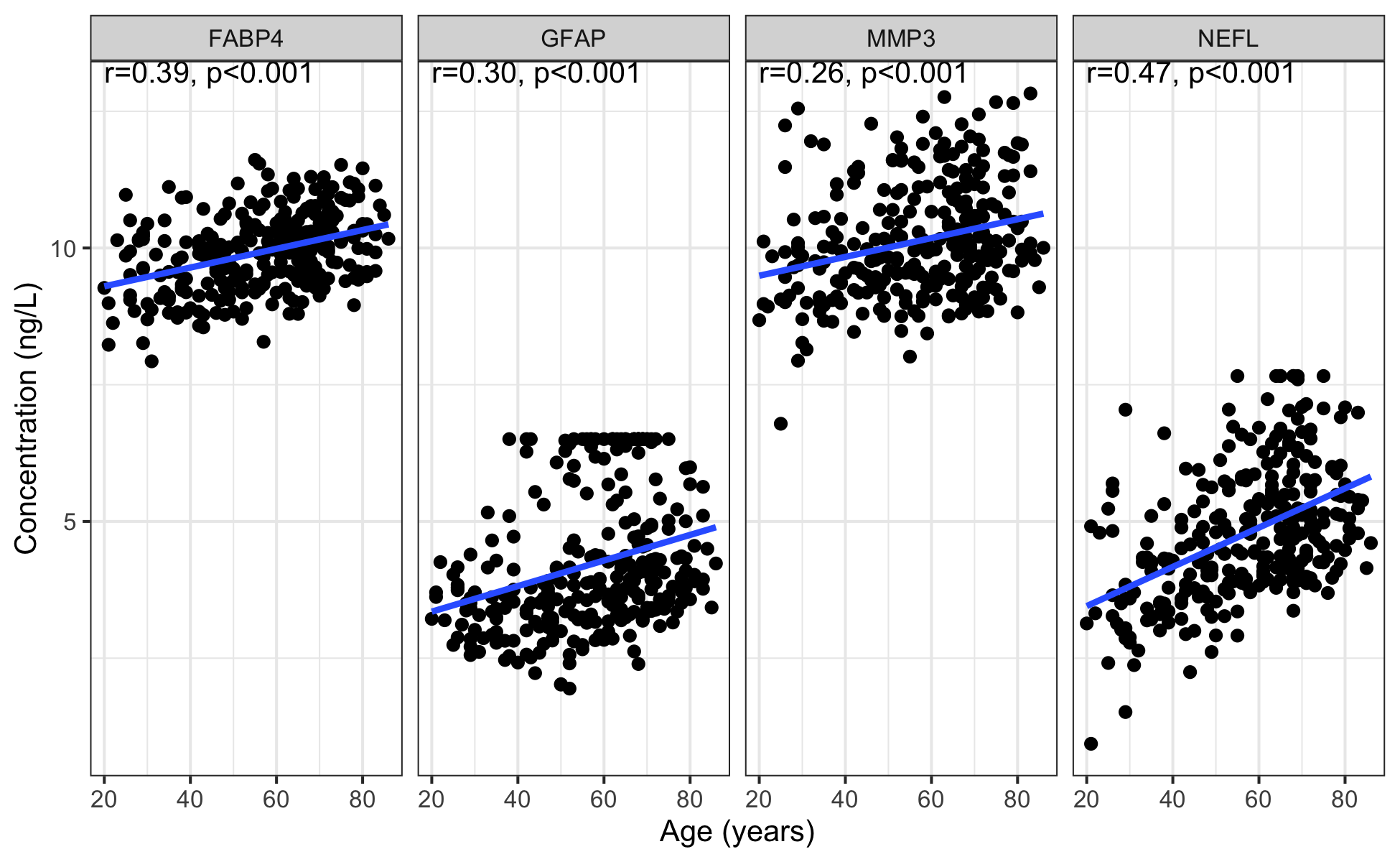
*

**Supplementary Figure 4**. Effect of age on the plasma concentrations of the four biomarkers shown (FABP4, GFAP, MMP3, NEFL) for the 291 patients with age information. r is the Spearman correlation coefficient. In general, the plasma concentration increases with age, for all four proteins.


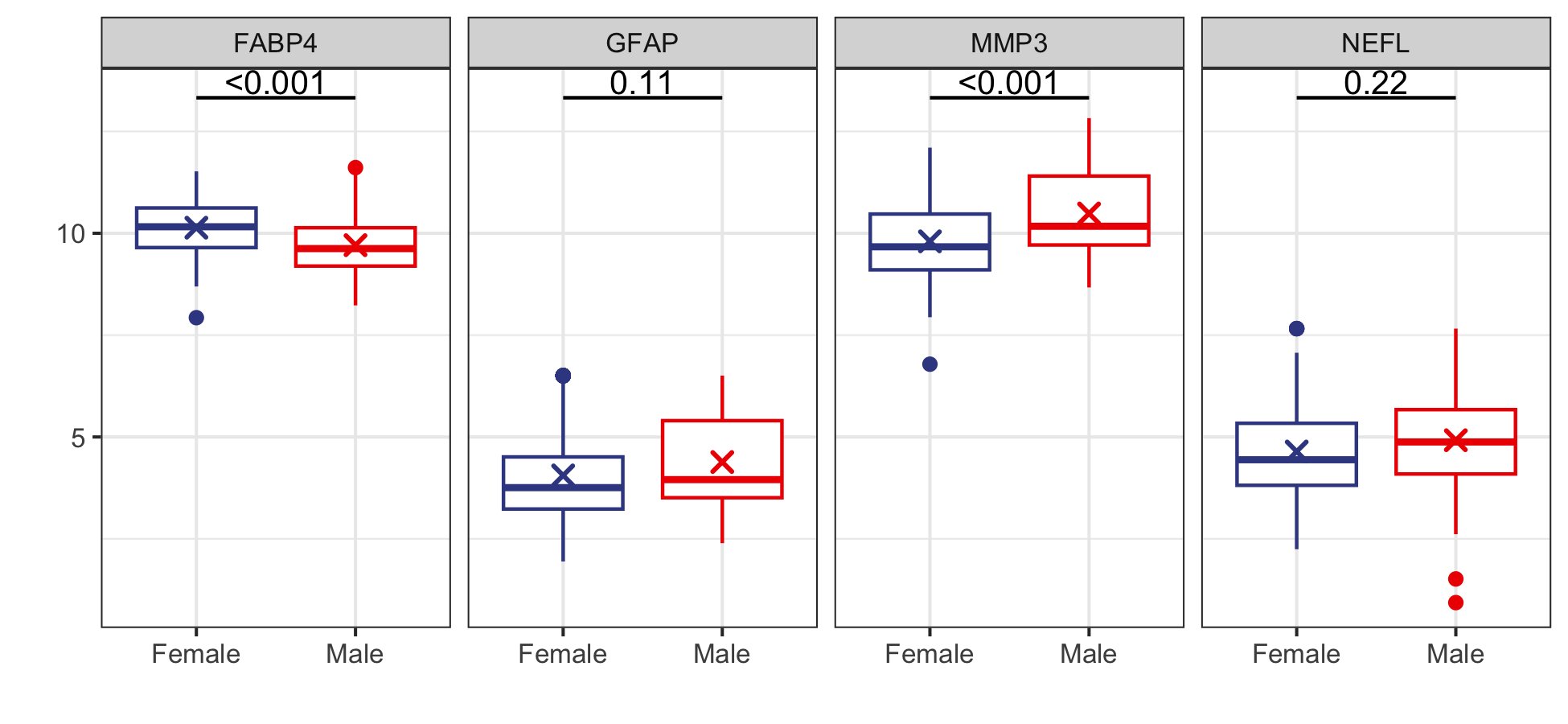


**Supplementary Figure 5**. Effect of sex on plasma values of the four shown biomarkers (FABP4, GFAP, MMP3, NEFL). In general, males have higher plasma values of the biomarkers than females, except for FABP4. Boxplots indicate median (horizontal lines) and interquartile range. Mean is marked with an ‘x’. The significance level of independent t-tests is shown at the top of each plot.


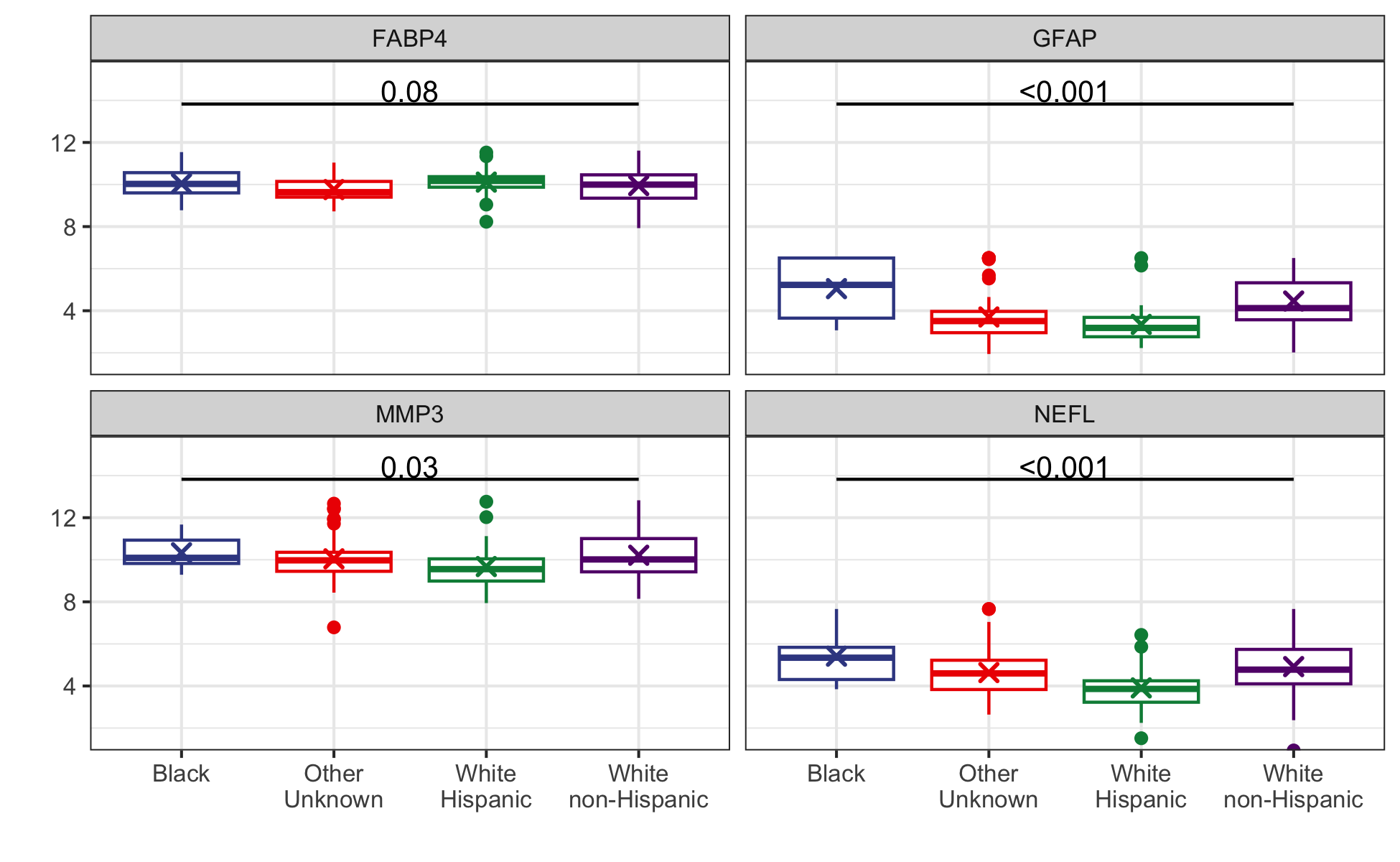


**Supplementary Figure 6**. Effect of ethnicity on plasma values of the four shown biomarkers (FABP4, GFAP, MMP3, NEFL). Boxplots indicate median (horizontal lines) and interquartile range. In general, blacks have higher plasma values of the biomarkers, and white Hispanics the lowest, but the differences are relatively small. The significance of ANOVA tests of mean differences is shown at the top of each plot. Mean values are indicated with ‘x’.


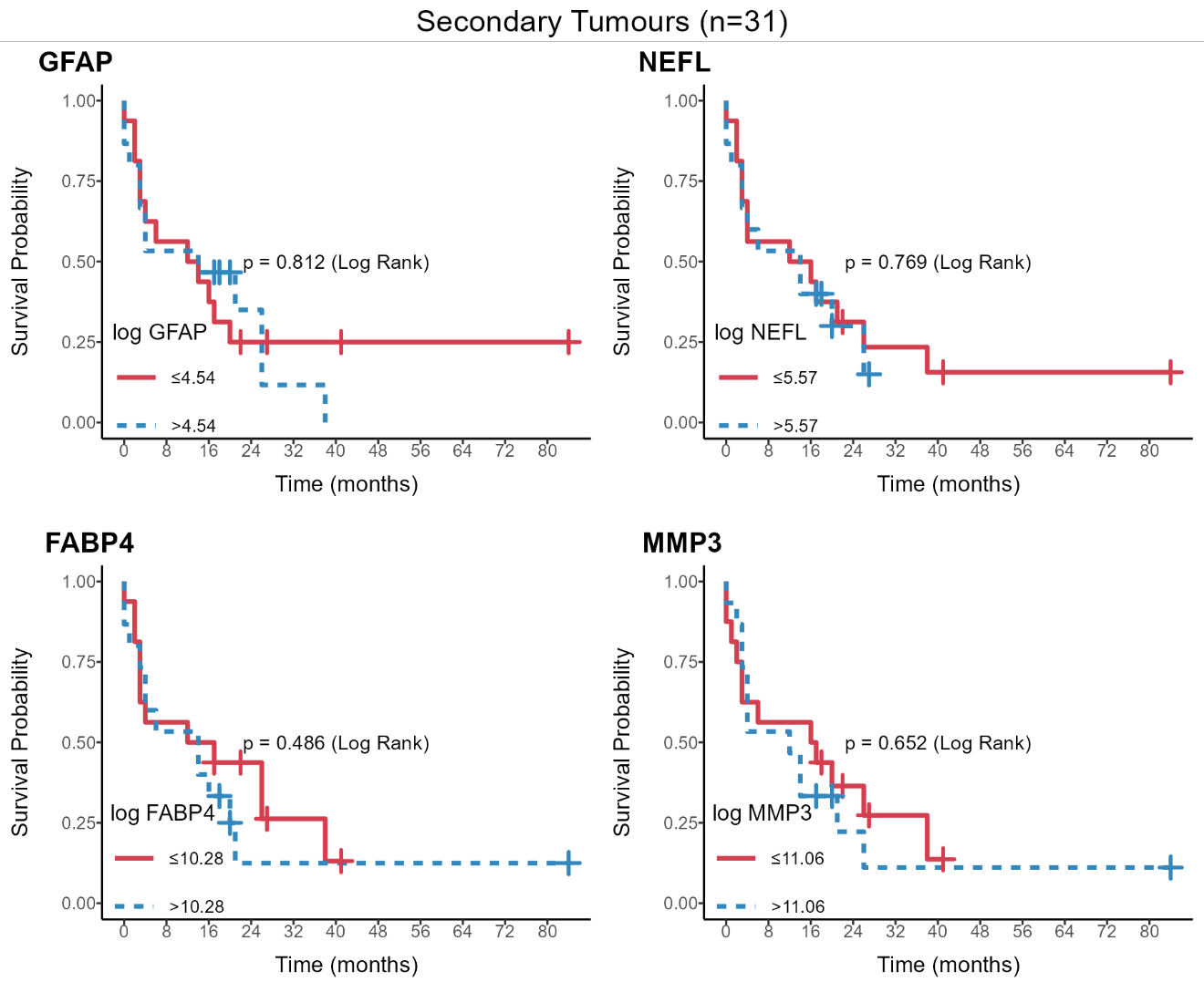


**Supplementary Figure 7**. Survival analysis (Kaplan-Meier plots) of patients with secondary metastases divided into high (blue crosses) or low (red crosses) plasma GFAP, NEFL, MMP3 and FABP4. The median of each protein concentration was used as a cut-off. Cut-offs are shown after log transformation, along with the p value, calculated by the log-rank test. None of the proteins are associated with overall survival. in these patient subgroups


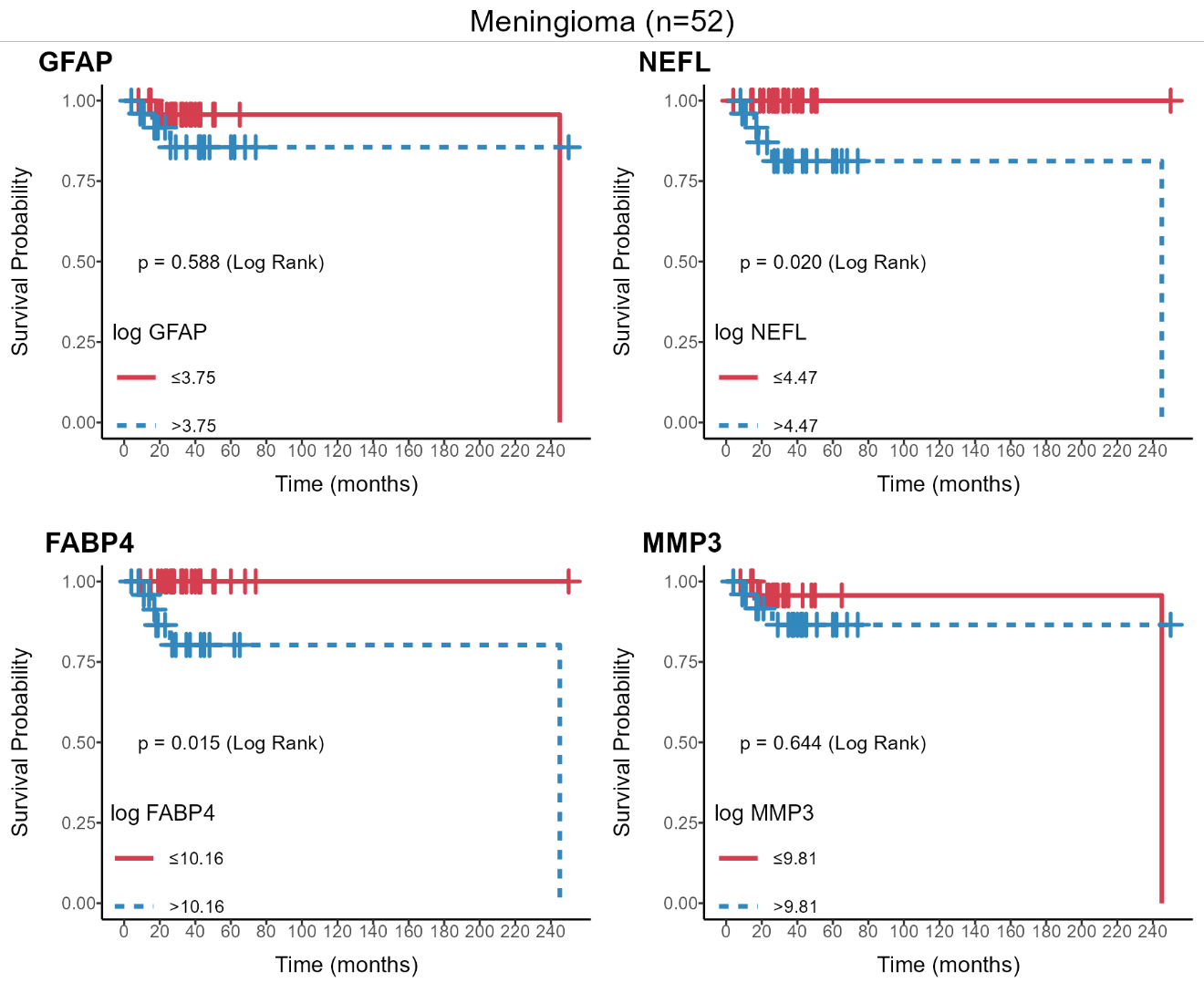


**Supplementary Figure 8**. Survival analysis (Kaplan-Meier plots) of patients with meningioma divided into high (blue crosses) or low (red crosses) plasma GFAP, NEFL, MMP3 and FABP4. The median of each protein concentration was used as a cut-off. Cut-offs are shown after log transformation, along with the p value, calculated by the log-rank test. Only FABP4 and NEFL are weakly, but significantly associated with meningioma patient survival.

*
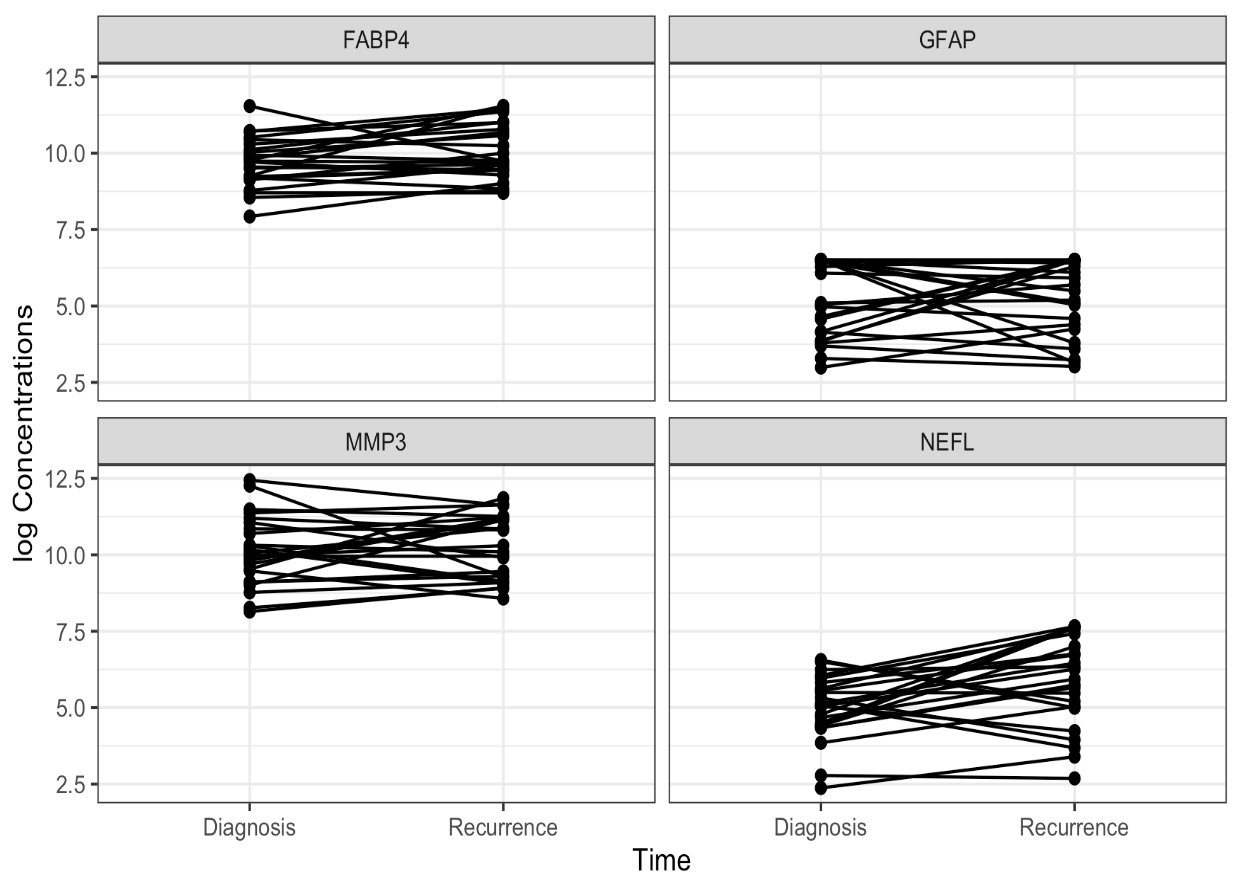
*

**Supplementary Figure 9**. Biomarker plasma concentrations of FABP4, GFAP, MMP3, and NEFL at diagnosis and at the time of recurrence, for 24 patients. See text for more detailed comment.

**Supplementary Table 1**. Categories of patients with astrocytomas, oligodendrogliomas and meningiomas. Shown are the number of patients by diagnosis (pre 2021 and post 2021 WHO classification).

| **pre 2021 WHO classification** | **IDH1 Status** | **1p/19q co-deletion** | **2021 WHO classification** | **Meningioma (n=70)** | **Glioma (n=126)** |
| --- | --- | --- | --- | --- | --- |
| Anaplastic Astrocytoma, grade 3 | Wild Type | no | GBM wild-type IDH |  | 8 |
| Anaplastic Astrocytoma, grade 3 | Mutated | no | Astrocytoma IDH mut no 1p19q co-deletion |  | 11 |
| Astrocytoma, grade 2 | Mutated | no | Astrocytoma IDH mut no 1p19q co-deletion |  | 11 |
| Astrocytoma, grade 2 | Wild-type | no | GBM wild-type IDH |  | 2 |
| GBM | Wild-type | no | GBM wild-type IDH |  | 67 |
| GBM | Mutated | no | Astrocytoma IDH mut no 1p19q co-deletion |  | 4 |
| Oligodendroglioma, grade 2 | Mutated | yes | Oligodendroglioma IDH mut/1p19q co-deletion |  | 10 |
| Anaplastic Oligodendroglioma, grade 3 | Mutated | yes | Oligodendroglioma IDH mut/1p19q co-deletion |  | 13 |
| Meningioma, grade 1 |  |  | Meningioma | 45 |  |
| Atypical Meningioma, grade 2 |  |  |  | 25 |  |

**Supplementary Table 2**. Demographic characteristics of the patients (sd, standard deviation). Categorization was done with the old and the new WHO criteria.

| **Patient Characteristics (n=291)** |  |
| --- | --- |
|  | N (%) |
| **Age at time of collection** |  |
| Mean (sd) | 56.9 (15.9) |
| Median (Min, Max) | 60 (20, 86) |
| **Sex** |  |
| Female | 152 (52) |
| Male | 139 (48) |
| **Ethnic Category** |  |
| Black | 10 (3) |
| Other / Unknown | 59 (20) |
| White, Hispanic | 30 (10) |
| White, non-Hispanic | 192 (66) |
| **Category** |  |
| GBM | 77 (26) |
| Astrocytoma mutant IDH no 1p19q co-deletion | 26 (9) |
| Oligodendroglioma mutant IDH 1p19q co-deletion | 23 (8) |
| Secondary tumors | 35 (12) |
| Meningiomas | 70 (24) |
| Schwannomas | 15 (5) |
| Pituitary adenomas | 15 (5) |
| Normal individuals | 30 (10) |
| **WHO grade** |  |
| grade 1 | 75 (26) |
| grade 2 | 48 (16) |
| grade 3 | 32 (11) |
| grade 4 | 106 (36) |
| not applicable | 30 (10) |

**Supplementary Table 3.** Effect of sex on the plasma concentration of the four biomarkers (sd, standard deviation).

| Covariate |  | Full Sample (n=291) | Female (n=152) | Male (n=139) | p-value |
| --- | --- | --- | --- | --- | --- |
| **log FABP4** |  |  |  |  | **<0.001** |
| Mean (sd) |  | 9.9 (0.7) | 10.1 (0.7) | 9.7 (0.7) |  |
| Median (Min, Max) |  | 10.0 (7.9, 11.6) | 10.2 (7.9, 11.5) | 9.6 (8.2, 11.6) |  |
| **log GFAP** |  |  |  |  | **0.02** |
| Mean (sd) |  | 4.2 (1.2) | 4.1 (1.2) | 4.4 (1.2) |  |
| Median (Min, Max) |  | 3.9 (1.9, 6.5) | 3.8 (1.9, 6.5) | 4.0 (2.4, 6.5) |  |
| **log MMP3** |  |  |  |  | **<0.001** |
| Mean (sd) |  | 10.1 (1.0) | 9.8 (0.9) | 10.5 (1.0) |  |
| Median (Min, Max) |  | 10.0 (6.8, 12.8) | 9.7 (6.8, 12.1) | 10.2 (8.7, 12.8) |  |
| **log NEFL** |  |  |  |  | **0.03** |
| Mean (sd) |  | 4.8 (1.2) | 4.6 (1.2) | 4.9 (1.2) |  |
| Median (Min, Max) |  | 4.6 (0.9, 7.7) | 4.4 (2.2, 7.7) | 4.9 (0.9, 7.7) |  |

**Supplementary Table 4**. Effect of ethnicity on plasma biomarker values (sd, standard deviation). All patients with ethnicity information were included.

| Covariate | Full Sample (n=291) | Black (n=10) | Other /  Unknown (n=59) | White,  Hispanic (n=30) | White,  non-Hispanic (n=192) | p-value |
| --- | --- | --- | --- | --- | --- | --- |
| **log FABP4** |  |  |  |  |  | 0.08 |
| Mean (sd) | 9.9 (0.7) | 10.0 (0.8) | 9.8 (0.5) | 10.1 (0.7) | 10.0 (0.7) |  |
| Median  (Min ,Max) | 10.0 (7.9, 11.6) | 10.0 (8.8, 11.5) | 9.6 (8.7, 11.0) | 10.2 (8.2, 11.5) | 10.0 (7.9, 11.6) |  |
| **log GFAP** |  |  |  |  |  | **<0.001** |
| Mean (sd) | 4.2 (1.2) | 5.1 (1.5) | 3.7 (1.1) | 3.3 (1.0) | 4.5 (1.2) |  |
| Median  (Min, Max) | 3.9 (1.9, 6.5) | 5.2 (3.1, 6.5) | 3.5 (1.9, 6.5) | 3.2 (2.2, 6.5) | 4.1 (2.0, 6.5) |  |
| **log MMP3** |  |  |  |  |  | **0.03** |
| Mean (sd) | 10.1 (1.0) | 10.3 (0.8) | 10.0 (1.0) | 9.7 (1.1) | 10.2 (1.0) |  |
| Median (Min, Max) | 10.0 (6.8, 12.8) | 10.1 (9.3, 11.7) | 10.0 (6.8, 12.7) | 9.6 (7.9, 12.8) | 10.0 (8.1, 12.8) |  |
| **log NEFL** |  |  |  |  |  | **<0.001** |
| Mean (sd) | 4.8 (1.2) | 5.4 (1.3) | 4.6 (1.1) | 3.9 (1.1) | 4.9 (1.2) |  |
| Median (Min, Max) | 4.6 (0.9, 7.7) | 5.3 (3.8, 7.7) | 4.6 (2.6, 7.7) | 3.9 (1.5, 6.4) | 4.8 (0.9, 7.7) |  |

**Supplementary Table 5**. Plasma protein values of the four biomarkers and relation to the listed genetic abnormalities. Most genetic abnormalities are associated with variations in biomarker protein plasma concentration in various patient groups (Grp).sd, standard deviation. For more definitions see text.

|  | Grp1/Grp2 | Grp1 Mean (sd) | Grp2 Mean (sd) | p-value (t-test) |
| --- | --- | --- | --- | --- |
| **FABP4** |  |  |  |  |
| IDH1 Status | Wild Type / Mutated | 9.85 (0.73) | 9.39 (0.66) | <0.001 |
| ATRX Expression | Retained / Lost | 9.82 (0.70) | 9.14 (0.63) | <0.001 |
| P53 NGS status | Wild Type / Mutated | 9.79 (0.74) | 9.48 (0.72) | 0.03 |
| EGFR Status | Wild Type / Mutated | 9.59 (0.70) | 10.17 (0.64) | <0.001 |
| MGMT Promotor Methylation | Positive / Negative | 9.52 (0.71) | 9.81 (0.75) | 0.03 |
| TERT Promoter | Mutated / Wild Type | 9.81 (0.75) | 9.47 (0.68) | 0.01 |
| CDKN2A B p16 | Wild Type / Lost | 9.61 (0.69) | 9.77 (0.80) | 0.27 |
| V1p 19q Co-deletion | Negative / Positive | 9.53 (0.73) | 9.55 (0.71) | 0.94 |
| NF1 | Wild Type / Mutated | 9.67 (0.77) | 9.90 (0.57) | 0.14 |
| **GFAP** |  |  |  |  |
| IDH1 Status | Wild Type / Mutated | 5.45 (1.29) | 3.81 (0.77) | <0.001 |
| ATRX Expression | Retained / Lost | 4.99 (1.40) | 4.06 (1.04) | <0.001 |
| P53 NGS status | Wild Type / Mutated | 5.02 (1.35) | 4.50 (1.39) | 0.05 |
| EGFR Status | Wild Type / Mutated | 4.48 (1.31) | 5.22 (1.35) | 0.03 |
| MGMT Promotor Methylation | Positive / Negative | 4.51 (1.32) | 5.12 (1.40) | 0.01 |
| TERT Promoter | Mutated / Wild Type | 5.18 (1.34) | 4.30 (1.28) | <0.001 |
| CDKN2A B p16 | Wild Type / Lost | 4.38 (1.28) | 5.48 (1.26) | <0.001 |
| V1p 19q Co-deletion | Negative / Positive | 4.72 (1.39) | 3.66 (0.51) | <0.001 |
| NF1 | Wild Type / Mutated | 4.84 (1.39) | 4.89 (1.45) | 0.88 |
| **MMP3** |  |  |  |  |
| IDH1 Status | Wild Type / Mutated | 10.43 (1.05) | 9.78 (1.02) | <0.001 |
| ATRX Expression | Retained / Lost | 10.26 (1.08) | 9.85 (1.11) | 0.10 |
| P53 NGS status | Wild Type / Mutated | 10.28 (1.08) | 9.99 (1.06) | 0.16 |
| EGFR Status | Wild Type / Mutated | 10.02 (1.00) | 10.25 (1.15) | 0.41 |
| MGMT Promotor Methylation | Positive / Negative | 10.00 (1.07) | 10.39 (1.07) | 0.04 |
| TERT Promoter | Mutated / Wild Type | 10.38 (1.00) | 9.90 (1.17) | 0.02 |
| CDKN2A B p16 | Wild Type / Lost | 10.00 (1.10) | 10.46 (1.01) | 0.02 |
| V1p 19q Co-deletion | Negative / Positive | 10.11 (1.10) | 9.70 (0.92) | 0.10 |
| NF1 | Wild Type / Mutated | 10.21 (1.12) | 10.18 (0.98) | 0.91 |
| **NEFL** |  |  |  |  |
| IDH1 Status | Wild Type / Mutated | 5.72 (1.15) | 3.88 (0.95) | <0.001 |
| ATRX Expression | Retained / Lost | 5.30 (1.33) | 3.87 (1.15) | <0.001 |
| P53 NGS status | Wild Type / Mutated | 5.29 (1.35) | 4.56 (1.45) | 0.009 |
| EGFR Status | Wild Type / Mutated | 4.49 (1.33) | 5.90 (1.07) | <0.001 |
| MGMT Promotor Methylation | Positive / Negative | 4.65 (1.46) | 5.36 (1.28) | 0.005 |
| TERT Promoter | Mutated / Wild Type | 5.42 (1.23) | 4.44 (1.49) | <0.001 |
| CDKN2A B p16 | Wild Type / Lost | 4.53 (1.33) | 5.74 (1.23) | <0.001 |
| V1p 19q Co-deletion | Negative / Positive | 4.96 (1.53) | 4.02 (0.76) | <0.001 |
| NF1 | Wild Type / Mutated | 4.98 (1.47) | 5.35 (1.26) | 0.28 |

**Supplementary Table 6**. Distribution of patients across WHO grade and glioma diagnostic category. Cells are number of patients. Most patients (n=67) were grade 4 GMB wildtype IDH and there are no grade 4 olidodendrogliomas.

| Diagnostic Category | WHO Grade 2 | WHO Grade 3 | WHO Grade 4 |
| --- | --- | --- | --- |
| Astrocytoma IDH mutation no 1p19q co-deletion | 11 | 11 | 4 |
| GBM wildtype IDH | 2 | 8 | 67 |
| Oligodendroglioma IDH mutation/1p19q co-deletion | 10 | 13 | 0 |

**Supplementary Table 7**. Summary of the proportion of variation in protein levels explained by various predictor sets. The goal was to explore the importance of WHO category and diagnostic group to variation in protein levels. For GFAP and NEFL grade and diagnostic category combined explain more than half of variation in protein levels. For all proteins, WHO grade explains more unique variation than diagnostic group.

|  | Proportion of variation (R^2^) in proteins explained by different multivariable models | | | |
| --- | --- | --- | --- | --- |
| Protein | Full Model:  Age+Sex+WHO grade + diagnostic category | Reduced Model: diagnostic category and WHO grade | Unique contribution of diagnostic group ( | Unique contribution of WHO Grade |
| FABP4 | 0.28 | 0.16 | 0.03 | 0.05 |
| GFAP | 0.60 | 0.57 | 0 | 0.22 |
| MMP3 | 0.30 | 0.16 | 0 | 0.07 |
| NEFL | 0.62 | 0.52 | 0.04 | 0.10 |
